# Just-in-Time: Statewide Preceptor Development Through Brief Asynchronous Modules

**DOI:** 10.7759/cureus.91534

**Published:** 2025-09-03

**Authors:** Kelly L Smith, Victoria Boggiano, Ben A Blomberg, Laura Rachal, Lindsay Wilson, Fei Chen, Eric Zwemer

**Affiliations:** 1 Family Medicine, University of North Carolina School of Medicine, Chapel Hill, USA; 2 Medicine/Geriatrics, University of North Carolina School of Medicine, Chapel Hill, USA; 3 Internal Medicine-Pediatrics, Tulane University School of Medicine, New Orleans, USA; 4 Anesthesiology, University of North Carolina School of Medicine, Chapel Hill, USA; 5 Pediatrics, University of North Carolina School of Medicine, Chapel Hill, USA

**Keywords:** continuing medical education (cme), distance learning/e-learning, faculty development online, learning modules, preceptorship

## Abstract

Introduction

Clinical teachers are vital to the quality of the trainee learning environment. However, many of them undertake teaching roles without formal training. Time constraints and geographic distance of preceptors from central academic campuses may be barriers to such training. The University of North Carolina (UNC) School of Medicine (SOM)’s Academy of Educators (AOE) aimed to address these gaps through the development of AOE Essentials online training modules.

Methods

AOE Essentials consists of three asynchronous, self-paced modules designed for clinical preceptors across six clinical sites in North Carolina. A diverse group of 22 faculty members developed the modules, which cover topics such as setting expectations, completing evaluations, and providing effective feedback. Each module includes embedded videos, diagnostic quizzes, and pre- and post-tests of satisfaction and knowledge gain for assessment. Knowledge gain in each module is assessed by four to five multiple-choice, one-best-answer questions for each quiz that were initially designed by each module’s faculty and subsequently reviewed by study authors with expertise in medical education scholarship generally and survey methodology specifically. Three separate questions assessing the quality/quantity of information and clarity of visual information were adapted from the Mobile App Rating Scale (MARS).

Results

To date, 254 modules have been completed by faculty. The Expectations module (n = 104) was completed at higher rates than Evaluations (n = 71) or Feedback (n = 79) models. Analysis revealed significant improvements in pre-post knowledge scores for the Expectations (p<0.001) and Evaluation (p<0.001) modules. The Feedback module showed no significant change (p = 0.398), though this may have been limited by the ceiling effect and potential prior knowledge in this area. Participant feedback indicated high satisfaction, with average ratings ranging from 4.47 to 4.7 for content relevance and clarity. Additionally, 97% of participants would recommend the modules to colleagues.

Conclusions

AOE Essentials represents a novel asynchronous, multi-site faculty development program specifically designed for geographically dispersed clinical preceptors, demonstrating measurable knowledge gains and high participant satisfaction. Limitations of this study include completion of the evaluation immediately after completion of the modules, thus only assessing immediate knowledge gains, as well as concerns about a possible ceiling effect for some participants, limiting the ability to determine knowledge acquisition during the module. Future initiatives will explore knowledge retention of participants at three to six months, creation of additional training modules, and the potential for mandatory training for preceptors. Despite its limitations, this program may serve as a template for other institutions facing similar challenges in faculty development.

## Introduction

Clinical teachers play a vital role in shaping the clinical learning environment, yet many begin teaching without formal training in educational techniques [1]. While the prior faculty development work of Siddiqui, Cohen, and Kuhn has included case-based learning and diagnostic reasoning, accessible methods to engage geographically dispersed faculty are lacking [2,3,4]. At the University of North Carolina (UNC) School of Medicine (SOM), medical students complete training at six clinical sites (Asheville, Chapel Hill, Charlotte, Wilmington, Raleigh, and Greensboro) across the state. Within these sites, some preceptors practice in even more rural locations. The UNC SOM Academy of Educators (AOE) is a collaboration of more than 400 faculty and residents across the SOM’s clinical sites who engage in teaching and mentorship [5]. The AOE provides opportunities for synchronous and asynchronous faculty development; however, not all opportunities are available to faculty across all clinical sites. Informal feedback from faculty noted that traditional noon-based live sessions (even with virtual options) were not conducive to their busy practice settings. Therefore, AOE Essentials was created to provide brief yet comprehensive, asynchronous, self-paced online modules that review essential skills for clinical preceptors.

While other institutions have previously described similar initiatives, these tools often are not accessible after scheduled training sessions [2,6,7]. This paper will describe the development, implementation, and evaluation of AOE Essentials to meet the learning needs of independent, busy clinical preceptors across the state. We hypothesized that by creating easily accessible, asynchronous online modules on high-yield teaching topics, clinical educators would be able to efficiently acquire teaching skills relevant to their work. The end goal was to improve the learning environment for medical students at different campuses and sites across the state, as recent studies suggest that adapting the curriculum to local student preferences may enhance student engagement [8].

This article was previously presented at the Association of American Medical Colleges 2024 Annual Academies Collaborative Meeting on November 8, 2024.

## Materials and methods

Module development and distribution

AOE Essentials is a faculty development package that reviews clinical teaching basics. Twenty-two faculty from three sites and 13 departments collaborated on module development. The initial package included three modules (Expectations, Evaluations, and Feedback) with embedded videos created using the platform Articulate Rise 360. Each module takes approximately 15 minutes to complete (https://linktr.ee/example_AOE_essentials). Standardized instructions and templates on module creation were provided to each group to promote consistency in content creation across sites. Each module group met iteratively to review current educational literature and selected important and common educational frameworks: The One Minute Learner [9] (Expectations), Ask-Tell-Ask Method [10] (Feedback), and RIME [11,12] and Task-Gap-Action (TGA) [13] (Evaluations). Each module included paired videos demonstrating suboptimal and best-practice teaching encounters. The asynchronous modules were shared with educational leadership at each site to distribute to faculty preceptors.

Evaluation

Pre- and post-quizzes (see Appendix) for learners’ self-assessment, satisfaction, and knowledge gains were administered. The four to five multiple-choice, one-best-answer questions for each quiz were initially designed by each module’s faculty to ensure alignment with the learning objectives and content. Questions then underwent review by study authors with expertise in medical education scholarship generally and survey methodology specifically. The quiz questions did not undergo pilot testing otherwise. Three separate questions assessing the quality/quantity of information and clarity of visual information were adapted from the Mobile App Rating Scale (MARS) [14], which has previously been used in the literature to evaluate the quality of mobile applications focused on education of both patients and providers [15,16]. We selected these items to specifically address the usability and engagement of the modules. Lastly, respondents were asked whether they would recommend the module to a colleague. The combination of these questions serves to assess both the Reaction and Learning levels of Kirkpatrick’s model for skill acquisition [17]. This study received exemption by the UNC Institutional Review Board 24-0214 on February 14, 2024. 

Data analysis

Normality check of the quiz score distribution was performed using visual assessment of Histogram and Q-Q plots, as well as the formal Shapiro-Wilk Test. Given the non-normal distribution of the quiz scores, we applied a two-sample Wilcoxon test to compare the differences in pre- and post-learning scores for each module. To account for multiple comparisons across the three modules, p-values were adjusted using the False Discovery Rate (Benjamini-Hochberg) correction method to control the expected proportion of false discoveries at α = 0.05. Additionally, 95% confidence intervals (95%CI) for the difference in location parameters (medians) were calculated using the Hodges-Lehmann estimator, which provides a non-parametric estimate of the median difference between pre- and post-surveys. Rank Biserial Correlation (r) was calculated as the effect size measure for each Wilcoxon test. These analyses were completed using R (R Core Team, 2021). 

To track the geographic location of module completion, we used IP addresses of completed post-module surveys to create a map of use in and around North Carolina.

## Results

**Figure 1:**
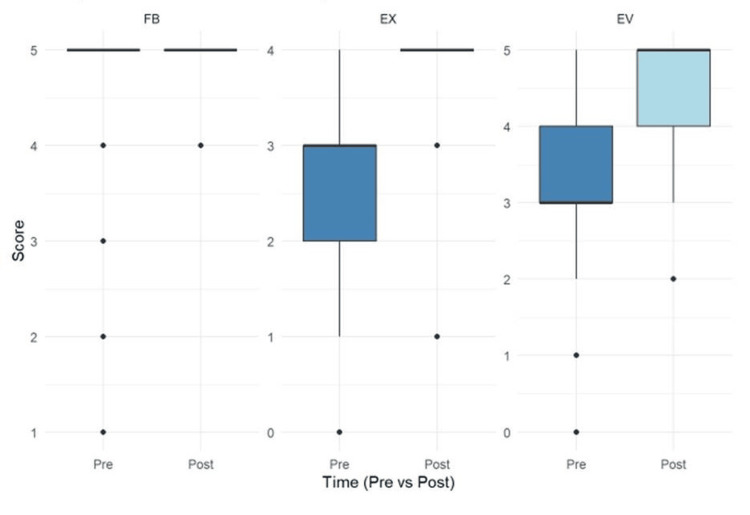
Boxplot of module quiz zcores The Feedback and Evaluation Modules each contain five questions, with a maximum possible score of 5; the Expectations Module contains four questions, with a maximum possible score of 4 FB: Feedback module; EX: Expectations module; EV: Evaluation module

To date, faculty members have completed 254 total modules. The Expectations module (n=104) was completed at higher rates than Evaluations (n=71) or Feedback (n=79). Quiz scores significantly increased after completing the Expectations (p<0.001, median difference 95% CI = (1,1), effect size (r) = 0.668) and Evaluation (p<0.001, median difference 95% CI = (1,2), effect size (r) = 0.695) modules. The change in scores on the Feedback module was not significant (p = 0.398, median difference 95% CI = (0,0), effect size (r) = 0.056). Table 1 summarizes the descriptive statistics (e.g., sample size, mean, standard deviation [SD], median, and interquartile range [IQR]), and Figure 1 shows boxplots of the pre- and post-learning quiz scores.

**Table 1:** Descriptive statistics of the scores by module SD: standard deviation; IQR: interquartile range

	Group	N	Mean	SD	Median	IQR
Feedback (FB)	Pre	79	4.67	0.78	5.00	0.00
	Post	63	4.84	0.37	5.00	0.00
Expectations (EX)	Pre	104	2.81	0.90	3.00	1.00
	Post	79	3.84	0.54	4.00	0.00
Evaluations (EV)	Pre	71	3.23	1.10	3.00	1.00
	Post	65	4.55	0.66	5.00	1.00

Faculty ratings for Module Quality, Quantity, and Visual Clarity can be seen in Table 2. Overall, each module was rated highly for each attribute, with all mean ratings >4.47/5 and all median ratings 5/5. Between 96.9% and 98.4% of participants indicated they would recommend each specific module to their colleagues.

**Table 2:** Faculty ratings of module Information Quality, Information Quantity, and Visual Clarity SD: standard deviation

	Group	Mean (range: 1-5)	SD	Median
Feedback (FB)	Content	4.7	0.58	5
	Extent of Information	4.65	0.56	5
	Visual Clarity	4.66	0.56	5
Expectations (EX)	Content	4.52	0.64	5
	Extent of Information	4.59	0.68	5
	Visual Clarity	4.62	0.58	5
Evaluations (EV)	Content	4.47	0.62	5
	Extent of Information	4.62	0.58	5
	Visual Clarity	4.63	0.49	5

**Figure 2:**
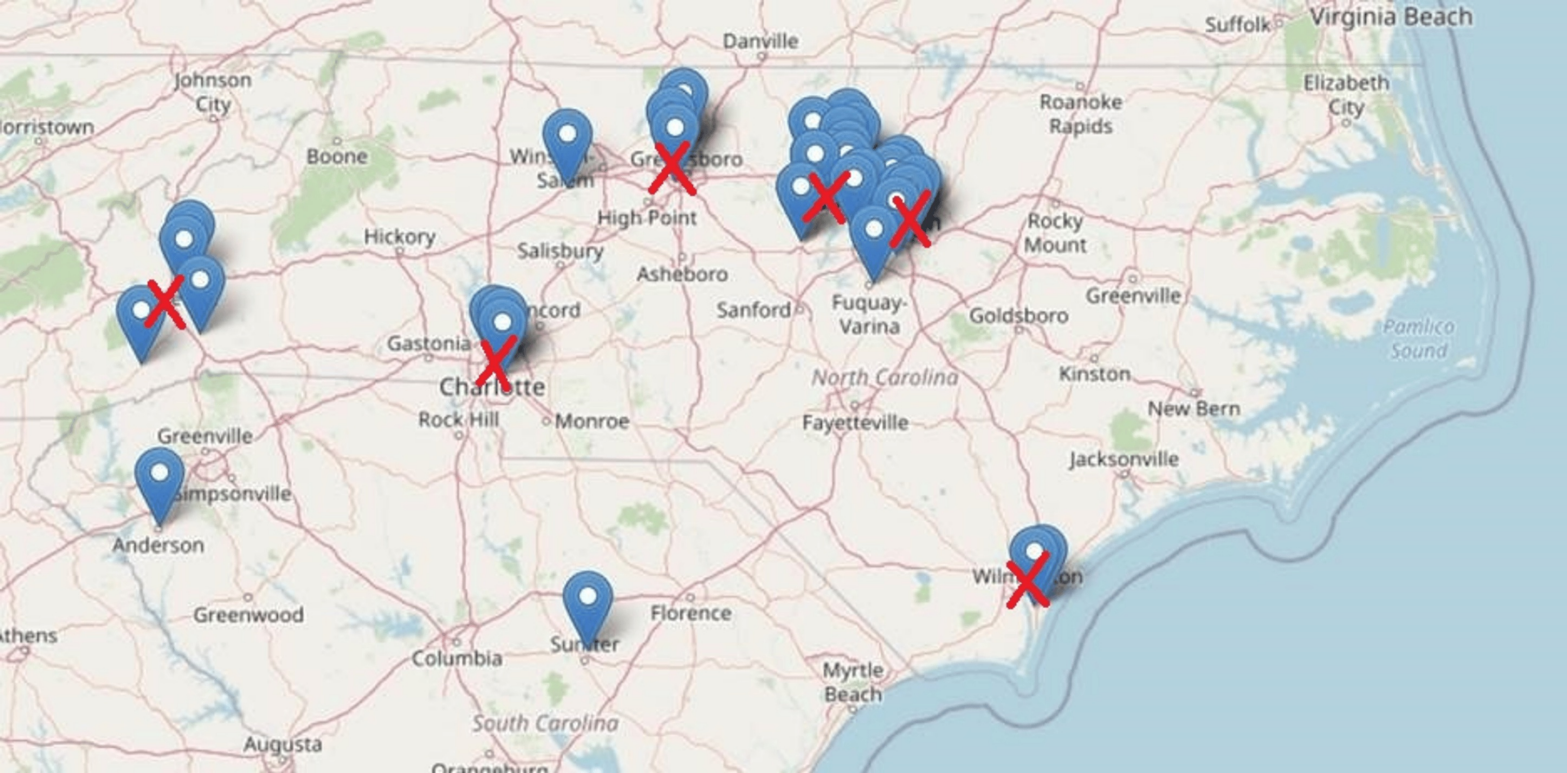
Map of AOE Essentials use in and around North Carolina Red “X”: Location of clinical training site. Pin: Completion of the AOE Essentials module by one user

The map in Figure 2 demonstrates uptake at the six clinical training sites for UNC medical students: Asheville, Chapel Hill, Charlotte, Greensboro, Raleigh, and Wilmington. Notably, we also saw an uptake at several other locations in and around North Carolina, suggesting more dispersed uptake depending on clinical preceptor location at the time of participation. 

## Discussion

The AOE Essentials address a longstanding challenge in medical education - equipping busy and geographically dispersed faculty with brief and accessible training on educational best practices. Two modules - Expectations and Evaluations - resulted in immediate gains in learning. The third - Feedback - did not demonstrate a significant increase in learning, likely because many participants received full marks on the pre-quiz, reflecting a ceiling effect, prior knowledge in this area, possible limitations in question design, and pre-existing faculty competence due to prior exposure to teaching on feedback.

AOE Essentials modules were rated similarly to other faculty development tools in the literature, including Tuesday’s Teaching Tips [18] and Special Study Modules (SSMs) [19] in Cross-Sectional Anatomy, which offer content efficiently and address barriers such as time constraints. Our module adds to existing offerings through its accessibility and asynchronous format, the relevance of the specific topics covered to teaching in an academic medical setting, and the use of pre- and post-questions that allow for demonstration of knowledge acquisition. 97% of participants who used the AOE Essentials modules reported that they would recommend these modules to a colleague. In addition, participants rated the visual and information quality and quantity of the modules highly. This emphasizes the value of structured, accessible, and modular learning approaches in enhancing teaching effectiveness and learner engagement across distributed educational settings.

There were more participants for the Expectations module than for the Feedback and Evaluations modules. This may be related to participants feeling that their skills in this area were lacking. Attrition between pre- and post-testing was significant across all modules. In the Feedback module, only 80% of participants completed both the pre- and post-surveys. In the Evaluation module, this rate improved to 92%, whereas in the Expectations module, it declined to 76%. Completion of the pre-survey was required to access the module; however, the post-survey was optional, allowing participants to forgo it.

Several limitations should be noted. First, the short video modules, while comprehensive, could not cover all the intricacies of the featured topics. We made every effort to ensure that each module was applicable to teaching undergraduate medical learners, but other topics may have been more relevant. Certain topics may also have been more pertinent to faculty at individual sites; however, it was not feasible to tailor content creation to specific locations. Second, our evaluation tools were completed immediately after the modules and therefore only assessed short-term knowledge gains. Although faculty were given permanent access to the modules for review, a valuable future direction would be to reassess participants several months or years later to evaluate knowledge retention. Third, the survey did not collect demographic information such as preceptors’ teaching experience, which could provide insights into the impact of the modules and the generalizability of our findings. Additionally, no comparison group was included. Another limitation may be that some pre-test questions were too easy, which could explain the ceiling effect observed in the Feedback module. Future iterations should consider revising the difficulty of quiz items. Lastly, our study is limited by self-selection bias, as participants who chose to complete the modules may not be representative of the broader faculty cohort.

Despite these limitations, the modules offer a practical, scalable approach to equipping distributed faculty with key teaching skills. It is important to note that in order to replicate AOE Essentials at other institutions, there needs to be a faculty champion with dedicated time to work with other faculty to create the modules. Future studies will explore retesting participating faculty 3-6 months after module completion to determine if their knowledge gains persist. An important next step will be assessing learner outcomes based on faculty participation in AOE Essentials. Therefore, future studies will attempt to link learner evaluations of faculty with module completion to see if there is a positive association. Other AOE Essentials modules are also being created on a range of clinical topics, including the struggling learner, procedural teaching, and fostering a growth mindset. Finally, to ensure a baseline level of educational training, we are exploring whether these modules can be part of mandatory onboarding for anyone precepting UNC medical students [20].

## Conclusions

AOE Essentials provides an example of how to teach faculty from multiple sites statewide about a variety of teaching pearls using asynchronous methods. The faculty reported that ease of use and content design were effective and led to excellent ratings. In addition, geographic uptake across the state of North Carolina is clearly demonstrated. Future studies will focus on assessing the impact of the modules on learners after faculty participation in AOE Essentials.

## Appendices

Evaluations pre-quiz

Q1. Which of the following is NOT an element of the Task-Gap-Action method of evaluation:

The learner and the faculty need to know the learning goal

The learner's performance should be compared with a standard

The faculty and learner are able to analyze complex events

An action plan should be developed with the learner to help close the performance gap

Q2. What does R-I-M-E stand for?

Recording, interpersonal communication, management, engagement

Reporter, interpreter, manager, expert

Recorder, interpreter, mastery, engager

Reporter, interpreter, manager, educator

Q3. How do medical students' and residents/fellows' evaluation forms differ?

They do not differ. They are the same.

Resident/Fellow evaluation forms are specific to their department; medical students evaluation forms are the same across all departments.

Resident and Fellow evaluation forms affect their ability to promote to more senior years of training, medical student forms are used only for feedback and do not count towards their rotation grade.

Resident and Fellow evaluation forms are for feedback only and do not affect their promotion to more senior years of training; medical student forms are used for feedback and count towards their rotation grade.

Q4. You are filling out a medical student evaluation you have worked with for a month and notice they are behind the standard for taking a medical history. On the evaluation form, you should:

Mark the score on the history-taking portion of the evaluation form

Mark the score on the history-taking portion of the evaluation form and put the knowledge gap the student has in the formative comments

Mark the score on the history-taking portion of the evaluation form and put the action items for improvement in the formative comments section

Mark the score on the history-taking portion of the evaluation form, then place the knowledge gap the student has and action items to improve this in the formative comments section

Mark the score on the history-taking portion of the evaluation form and tell the student via direct feedback, “you need to improve your history-taking skills.”

Q5. You evaluate a student who worked with you 2 half-days days in the clinic. You should:

Fill out the complete evaluation form

Only leave feedback in the comments section

Not fill out the evaluation form because too many are "insufficient observation or n/a"

Fill out the sections of the evaluation you observed and can evaluate fully; mark "insufficient observation or n/a" for the others

Q6 Educators who complete all modules will receive a certificate of completion. Please provide your email address for tracking for the certificate: __________

Evaluations post-quiz

Q1. Which of the following is NOT an element of the Task-Gap-Action method of evaluation:

The learner and the faculty need to know the learning goal

The learner's performance should be compared with a standard

The faculty and learner are able to analyze complex events

An action plan should be developed with the learner to help close the performance gap

Q2. What does R-I-M-E stand for?

Recording, interpersonal communication, management, engagement

Reporter, interpreter, manager, expert

Recorder, interpreter, mastery, engager

Reporter, interpreter, manager, educator

Q3. How do medical students' and residents/fellows' evaluation forms differ?

They do not differ. They are the same.

Resident/Fellow evaluation forms are specific to their department; medical students' evaluation forms are the same across all departments.

Resident and Fellow evaluation forms affect their ability to promote to more senior years of training; medical student forms are used only for feedback and do not count towards their rotation grade.

Resident and Fellow evaluation forms are for feedback only and do not affect their promotion to more senior years of training; medical student forms are used for feedback and count towards their rotation grade.

Q4. You are filling out a medical student evaluation you have worked with for a month, and notice they are behind the standard for taking a medical history. On the evaluation form, you should:

Mark the score on the history-taking portion of the evaluation form

Mark the score on the history-taking portion of the evaluation form, and put the knowledge gap the student has in the formative comments

Mark the score on the history-taking portion of the evaluation form and put the action items for improvement in the formative comments section

Mark the score on the history-taking portion of the evaluation form, then place the knowledge gap the student has and action items to improve this in the formative comments section

Mark the score on the history-taking portion of the evaluation form and tell the student via direct feedback, “you need to improve your history-taking skills.”

Q5. You evaluate a student who worked with you for 2 half-days in the clinic. You should:

Fill out the complete evaluation form

Only leave feedback in the comments section

Not fill out the evaluation form because too many are "insufficient observation or n/a"

Fill out the sections of the evaluation you observed and can evaluate fully; mark "insufficient observation or n/a" for the others

*Q6. Please list one practice change you intend to make as a result of this module.* ____________________________

*Q7. Please let us know one thing we should change about this module.* ______________________________________

*Q8. Please let us know one thing you liked about this module.* _________________________________________________

Q9. Quality of information: Is module content correct, well-written, and relevant to the goal/topic of the module?

Irrelevant/inappropriate/incoherent/incorrect

Poor. Barely relevant/appropriate/coherent/may be incorrect

Moderately relevant/appropriate/coherent/and appears correct

Relevant/appropriate/coherent/correct

Highly relevant, appropriate, coherent, and correct

Q10. Quantity of information: Is the extent of information covered within the scope of the module; and comprehensive but concise?

Minimal or overwhelming

Insufficient or possibly overwhelming

OK, but not comprehensive or concise

Offers a broad range of information, has some gaps or unnecessary detail; or has no links to more information and resources

Comprehensive and concise; contains links to more information and resources

Q11. Visual information: Is visual explanation of concepts - through charts/graphs/images/videos, etc. - clear, logical, correct?

Completely unclear/confusing/wrong or necessary but missing

Mostly unclear/confusing/wrong

OK but often unclear/confusing/wrong

Mostly clear/logical/correct with negligible issues

Perfectly clear/logical/correct

Q12. Would you recommend this module to your colleagues?

Yes

No

Q13. Educators who complete all modules will receive a certificate of completion. Please provide your email address for tracking for the certificate: _____________________________________________

Q14. For CME credit, please enter the following information.

Q15. Full Name ________________________________________________________________

Q16. Degree(s) ________________________________________________________________

Q17. Email Address ________________________________________________________________

Q18. PID (from UNC - if you do not have one, leave this blank) ________________________________________________________________
